# Epitaxial Growth Control of Crystalline Morphology and Electronic Transport in InSb Nanowires: Competition Between Axial and Radial Growth Modes

**DOI:** 10.3390/nano15181436

**Published:** 2025-09-18

**Authors:** Jiebin Zhong, Jian Lin, Miroslav Penchev, Mihrimah Ozkan, Cengiz S. Ozkan

**Affiliations:** 1 Aixtron Corporation, Santa Clara, CA 95054, USA; ryan.zhong@ymail.com; 2 Department of Mechanical and Aerospace Engineering, University of Missouri, Columbia, MO 65211, USA; linjian@missouri.edu; 3 Center for Environmental Research and Technology, University of California, Riverside, CA 92521, USA; mpenchev@cert.ucr.edu; 4 Electrical and Computer Engineering Department, University of California, Riverside, CA 92521, USA; 5 Mechanical Engineering Department, University of California, Riverside, CA 92521, USA

**Keywords:** InSb nanowires, epitaxy, III-V semiconductors, chemical vapor deposition, HRTEM, FET

## Abstract

This study investigates the morphological evolution of epitaxial indium antimonide (InSb) nanowires (NWs) grown via chemical vapor deposition (CVD). We systematically explored the influence of key growth parameters—temperature (300 °C to 480 °C), source material composition, gold (Au) nanoparticle catalyst size, and growth duration—on the resulting NW morphology, specifically focusing on NW length and tapering. Our findings reveal that the competition between axial and radial growth modes, which are governed by different growth mechanisms, dictates the final nanowire shape. An optimal growth condition was identified that yields straight and minimally tapered InSb NWs. High-resolution transmission electron microscopy (TEM) confirmed that these nanowires grow preferentially along the <110> direction, and electrical characterization via field-effect transistor (NW-FET) measurements showed that they are n-type semiconductors.

## 1. Introduction

Recently, semiconductor (e.g., Si and III-V) nanowires (NWs) have generated a continuously growing interest due to their intriguing physical properties and potential applications in nanoelectronics, optoelectronics, and biochemical sensors [1,2,3,4,5,6,7,8,9]. Silicon nanowires are being utilized for high-performance anodes [7] and for cutting-edge transistor fabrication at the 2 nm fabrication node [8]. Building Indium Gallium Arsenide (InGaAs) nanowire photodetectors directly onto silicon chips enables high-speed optical data reception, a key step toward creating fully integrated optical communication systems [9]. It has been previously mentioned that the parameters for controlled growth of NWs, including their diameter, morphology, growth direction, composition, and electrical properties, including conductivity and mobility, are crucial to characterize from the viewpoint of fundamental materials processing and the requirements for specific applications [10,11,12,13,14,15]. For example, the size-dependent quantum confinement effects and the application performance for nanoelectronics are significantly affected by the NW shape and morphology [16,17,18,19]. Au-catalyst-assisted epitaxial growth of III-V NWs has been widely studied by several research groups [20,21,22,23,24,25,26,27,28,29]. Results related to morphology, physical and electrical properties, and device performance vary among groups, and many growth-related aspects remain unclear, including the growth mechanisms under different conditions. Usually, the traditional molecular-impingement-induced vapor–liquid–solid (VLS) mechanism, first proposed by Wagner and Ellis in 1964, is assigned as the NW growth mechanism [30,31]. A later-proposed adatom-diffusion-induced VLS growth mode has been adopted, which has been the basis for postulating growth phenomena related to NW tapering at certain length scales [20,32,33]. However, most III–V NWs are grown at temperatures lower than the eutectic point for Au–group III materials, which disagrees with the VLS mechanism; therefore, the overall validity of the VLS mechanism for III–V NW growth is questionable [33]. An alternative is the vapor–solid–solid (VSS) mechanism—also described as the vapor–solid (VS) mechanism—which is also a potential candidate to describe the growth of III–V NWs. It suggests a solid particle assisting the growth rather than a liquid state, consequently resulting in NWs formed at lower temperatures [34,35]. All the same, the fundamentals of VSS and VS mechanisms are not well understood, and many controversies remain between VLS and VSS mechanisms [36,37]. In addition, migration of the Au catalyst during the growth process is reported [38], and Au may react with In [39,40]. Overall, the role of Au as the catalyst is still not fully understood. The complexity arises from the interplay of various factors, including the specific growth conditions and the behavior of the Au–In–Sb alloy. Overall, the NW growth process is complicated, and achieving repeatable growth and process control for shape and morphology requires a more detailed understanding of the growth modes and the exact role of the catalyst [39].

The morphology of the resulting NWs can be interpreted by the kinetics of the growth species involved and by diffusion of adatoms from the substrate and the Au catalyst [24,41,42,43]. NW morphology is mainly affected by different axial and radial growth under different processing conditions. Theoretical and experimental work has reported that a number of growth parameters impact the axial and radial growth of NWs, affecting the final morphology [24,25,26,27,28,29,32,44,45]. Fundamental experiments representing different growth conditions are a must for identifying the growth mechanism and achieving controlled growth of NWs for practical applications.

A member of the III-V semiconductor group, indium antimonide (InSb) is recognized as one of the most attractive materials for nano-building blocks for high-speed and low-power electronics [46], infrared detectors [47], and thermoelectric (TE) devices [48], attributed to its high bulk electron mobility (77,000 cm^2^ V^−1^ s^−1^ at 300 K), narrow direct band gap (0.17 eV at 300 K), and small effective mass [49,50,51]. InSb NWs have been synthesized using templated electrochemical deposition (ECD), metal–organic vapor-phase epitaxy (MOVPE), chemical beam epitaxy (CBE), molecular beam epitaxy (MBE), and chemical vapor deposition (CVD) [52,53,54,55,56,57]. Regarding controlled epitaxial growth of InSb NWs, recently, our group demonstrated the synthesis of sub-10 nm diameter InSb nanowires using epitaxial CVD [58]. Yang et al. reported single-crystalline InSb NWs synthesized via a three-zone CVD system [59,60]. Stoichiometry of InSb NWs via a vapor-phase transport was reported by Philipose et al. [61], and the effect of growth pressure on InSb NW growth was studied by Zhou et al. [61]. Although considerable efforts have been made on the optoelectronic applications of InSb NWs, the literature regarding the growth-control aspects for InSb NWs is still limited compared to other III-V materials. Without sufficient experimental studies, a complete understanding of InSb NW growth mechanisms and control of the growth process would be problematic. There has been limited information on the influence of growth parameters on epitaxial growth of InSb NWs and their morphology, which requires a study of the growth mechanisms [62].

In the current experimental work, we report on the Au-assisted growth of InSb NWs by CVD under different growth conditions and on changes in NW morphology depending on the growth parameters. Firstly, we investigated the effects of growth temperature and powder-source weight (corresponding to precursor vapor concentration) on the formation and morphology of InSb NWs. Au-assisted growth of InSb NWs was conducted at temperatures in the range of 330–450 °C and with different source material weights (5 mg, 20 mg, and 80 mg). Detailed morphological studies were carried out using scanning electron microscopy (SEM) and transmission electron microscopy (TEM). Statistical analysis shows that the NW length and the tapering factor are correlated with growth temperature and the amount of source material. We further studied the influence of growth time on the changes in NW morphology. Based on the evolution of NW morphology, we discuss a potential growth model for axial and radial growth modes that accounts for the kinetics of growth species or atoms, adatoms on the growth substrate, and diffusion of Au atoms in the catalyst. Finally, application-desired straight NWs with a low tapering factor were obtained by optimizing the growth parameters. Transport properties of InSb NWs were studied by fabricating NW field-effect transistors (NW-FET). Our findings provide a basis for understanding the process-dependent morphology of InSb NWs, which is applicable to studying other III–V NW systems as well.

## 2. Results and Discussion

### 2.1. Morphology of InSb NWs

#### 2.1.1. Effect of Temperature and Source Weight on InSb NW Morphology

**Figure 1:**
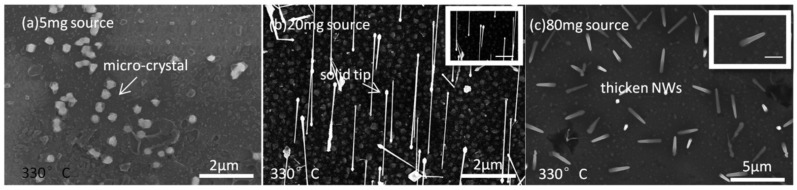
SEM images of InSb NWs grown on an InSb (100) substrate at 330 °C with different InSb powder-source amounts: (**a**) 5 mg; (**b**) 20 mg, showing solid tips atop the NWs; and (**c**) 80 mg, exhibiting thicker sidewalls. Insets in (**b**,**c**) are high-magnification SEM images of the corresponding samples. The growth duration was 60 min for all samples, and no NW growth was observed for the 5 mg source sample. Scale bars for the inset SEM images in (**b**,**c**) are 500 nm.

**Figure 2:**
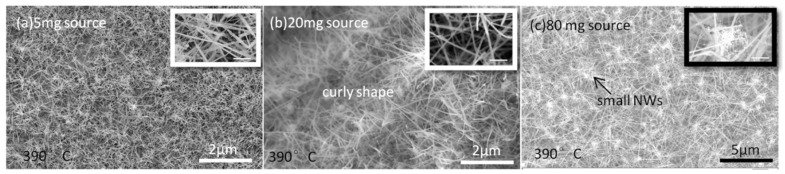
SEM images of high-density InSb NWs grown on an InSb (100) substrate at 390 °C with different InSb powder-source amounts: (**a**) 5 mg, showing rough NW bodies; (**b**) 20 mg, showing curly morphology; and (**c**) 80 mg, showing small, tangled NWs around the initially grown NWs. Insets in (**a**–**c**) are high-magnification SEM images of the corresponding as-grown samples. The growth duration was 60 min for all samples. Scale bars for the inset SEM images in (**a**–**c**) are 200 nm.

**Figure 3:**
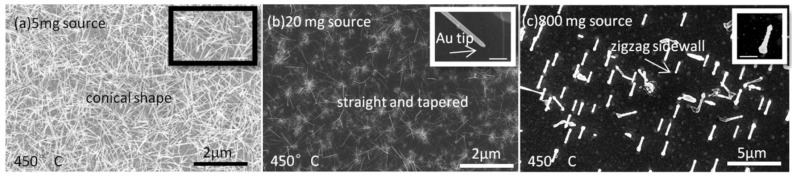
SEM images of InSb NWs grown on an InSb (100) substrate at 450 °C with different InSb powder-source amounts: (**a**) 5 mg, showing conical shapes with sharp tips; (**b**) 20 mg, showing straight, tapered morphology with round tips on top; and (**c**) 80 mg, showing zigzag NW sidewalls. InSb NWs grow epitaxially on InSb (100) with an axial 〈110〉 direction; on standard (100) wafers with a 〈110〉 major flat, the in-plane projection of the NW axis is parallel to the flat (axis 45° to the surface normal). Insets in (**a**–**c**) are high-magnification SEM images of the corresponding samples. The growth duration was 60 min for all samples. Scale bars for the inset SEM images in (**a**–**c**) are 200 nm.

It is well known that growth temperature and precursor pressure play important roles in controlling the growth of NWs, particularly their morphology [20,63]. Since a tube-in-tube CVD setup with a powder source as the precursor is widely used, it is crucial to evaluate the effect of powder-source amount on NW growth. InSb NW morphology is found to be influenced by the growth temperature in the range of 330–450 °C and by the amount of powder source (5 mg, 20 mg, and 80 mg), corresponding to the precursor vapor concentration (PVC). A series of SEM images of InSb NWs synthesized under different processing conditions are presented in Figure 1, Figure 2 and Figure 3.

In Figure 1, the InSb NWs shown were synthesized at 330 °C for 60 min using 5 mg (Figure 1a), 20 mg (Figure 1b), and 80 mg (Figure 1c) of powder source, respectively. Using a 5 mg powder source as the precursor (Figure 1a), only large bulk crystals are observed on the growth substrate. This is probably because NWs cannot be grown under low-PVC conditions at a low temperature (330 °C in this case), which is lower than the critical temperature allowing NW growth [34,45]. Similar conditions will lead to the growth of 2D layers or microcrystals on the substrate [21]. By increasing PVC by adding more powder, InSb NWs were successfully synthesized on the substrates, and the typical NW morphology is shown in Figure 1b,c. Using 20 mg of source material at 330 °C (Figure 1b), NWs indicate a larger tip diameter of about 100 nm and a smaller bottom diameter of about 50 nm, with a uniform growth direction, and the NW length is about 3.2 µm. A higher-magnification image inset in Figure 1b shows that the NWs are tapered gradually toward the base, and solid-shaped catalytic particles (CPs) are located on top of the NWs. This reverse tapering in NW morphology can be explained by Ostwald ripening (coarsening) of the catalyst particles during the annealing period, resulting in the formation of larger Au–In alloy particles [58,64].

Reverse tapering arises from Ostwald ripening and In alloying in the catalytic droplet: larger droplets yield wider tips and reverse-tapered bodies, while smaller droplets—depleted by ripening—can produce thinner or arrested NWs. Material for droplet coarsening derives from Au diffusion from smaller seeds and In uptake from the vapor/adatom flux. Shorter-length and larger-diameter NWs are observed in Figure 1c, corresponding to low growth temperatures and high-PVC conditions. Larger-diameter NWs probably occur due to growth species impinging directly on the NW sidewalls, resulting in significant radial growth. A 2D layer with a constant growth rate is also formed on the substrate, contributing to the shorter length of NWs [21,65,66].

The temperature dependence of NW morphology was also investigated. Figure 2a–c shows SEM images of InSb NWs grown at 390 °C with 5 mg, 20 mg, and 80 mg powder sources, respectively; other growth conditions were kept the same. Longer but curly shaped NWs with higher density on the growth substrate were obtained, indicating a higher growth rate compared to lower-temperature (330 °C) conditions. However, the NW growth direction is not clearly distinguished due to the inclination angle to the substrate. From Figure 2a, the NW diameter is in the range of 20–100 nm with uneven NW morphology, which might be attributed to an insufficient precursor supply. Curly NWs with a more uniform diameter are observed in Figure 2b. The curly shape implies that the growth kinetics change at the NW nucleation interface, leading to a continuous change in the contact angle and interfacial direction. Consequently, an unstable driving force results in the curly shape of NWs [64]. Figure 2c shows small, tangled InSb NWs around axially grown NWs. Kawashima et al. demonstrated that, because of the migration of catalyst Au atoms, smaller Si NWs were observed around the larger NW body [38]. Dick et al. reported that Au catalysts interacting with In-based NWs form nano trees when Au catalysts are deposited on NW sidewalls and excessive precursor is used [39]. In our case, Au catalyst migration might occur, but it is not significant at 390 °C. Therefore, under high-PVC conditions, migrated Au catalysts can interact with excess source material along the NW body. Consequently, they serve as catalysts, forming smaller NWs around the sidewalls. Such excess source material could derive directly from the vapor or from adatoms diffusing from the substrate near the NWs. In addition, the curly shape of NWs in Figure 2b can be further explained by Au catalyst migration out of the CPs, with adatoms diffusing toward the nucleation interface and incorporating with In atoms from the growth species. This dynamic process renders the nucleation interface unstable, resulting in the formation of curly shaped NWs. Figure 1 and Figure 2 indicate that Au catalyst migration and nucleation on NW sidewalls should be considered when evaluating the growth mechanism; details will be further discussed in the following section.

InSb NWs grown at 450 °C are shown in Figure 3. Conical-shaped InSb NWs are observed in Figure 3a under low-PVC conditions. No Au–In alloy particle tips are observed atop the NWs. Instead, the presence of a sharp tip and the high slope of the NW body suggest that Au atoms migrate out of the catalytic Au–In particles, resulting in the shrinking of the CPs, which finally disappear [42]. NWs with straight but tapered morphology are shown in Figure 3b. From the inset SEM image, the NW diameter is about 40 nm at the tip and 60 nm at the base. A shrinking, round tip is clearly observed at the top, which suggests a vapor–liquid–solid (VLS) mechanism. However, the catalyst size is smaller than 60 nm, likely due to the migration of Au atoms during growth. In Figure 3c, NWs grown with the largest source amount (80 mg) are tapered gradually toward the tip with a zigzag morphology along the NW sidewalls. The diameter at the base is 2–3 times larger than that of the tip. The zigzag shape of NWs suggests direct deposition onto the NW sidewalls from the vapor source or from adatoms on the substrate. Such zigzag sidewalls are usually observed at high temperatures and high-PVC conditions [63,67].

#### 2.1.2. Growth Time Dependence of InSb NW Morphology

**Figure 4:**
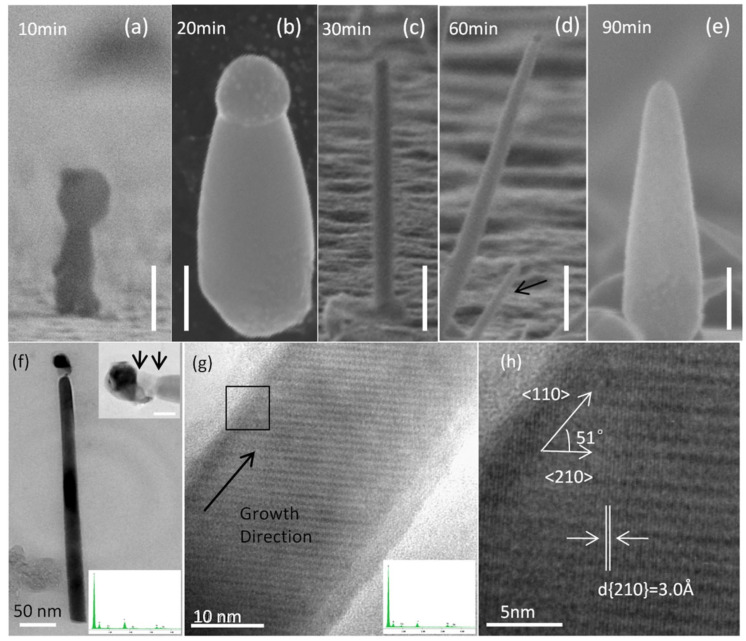
SEM images illustrating the morphology of InSb NWs grown after (**a**) 10, (**b**) 20, (**c**) 30, (**d**) 60, and (**e**) 90 min. The growth temperature was 450 °C, and a 20 mg powder source was used. Panels (**a**,**e**) are viewed along the surface normal; (**b**–**d**) are viewed at 45° from the normal, and (**b**) was rotated by 135° for better comparison with the other images. Scale bars are 100 nm for (**a**,**b**), and 500 nm for (**c**–**e**). (**f**) TEM image of a typical tapered NW. The inset at the top right is a higher-magnification TEM of the catalytic particle; the NW neck is marked between two arrows. The scale bar is 20 nm. The inset at the bottom right shows the EDAX measurement at the catalytic particle region (see Figure S4 for a detailed high resolution EDAX spectrum). (**g**) HRTEM image of the NW, indicating the growth direction. The diffraction pattern in Figure S4 indicates the presence of the {220} family of planes of the InSb zincblende structure. (**h**) A magnified rectangular section of the HRTEM image in (**g**). The white line markings show a measured d spacing of 3.0 Å, indicating a {210} plane. An angle of 51° between the growth direction and 〈210〉 indicates a 〈110〉 growth direction of the NW.

It has been observed that Au catalyst atoms may migrate during the growth process, based on the experiments described above. To clarify the effect of Au catalyst migration on NW morphology and to better understand the entire NW growth process, a series of samples was synthesized for growth durations of 10, 20, 30, 60, and 90 min at a 450 °C growth temperature (to enable a high Au catalyst migration rate) using a 20 mg powder source. Figure 4a–e show SEM images for these five samples, respectively. NWs grown for 10 min (Figure 4a) are highly tapered with a wide base, and approximately 50 nm diameter Au–In CPs are observed at the tips. The NW body displays a rough morphology, and not all catalyst particles have NWs grown underneath. Figure 4b shows an NW grown for 20 min with much smoother sidewalls. Round-shaped CPs are clearly seen with a diameter of approximately 80 nm, slightly larger than the original catalyst particles, which can be attributed to alloying with indium. Straight InSb NWs with a uniform diameter are observed in the sample grown for 30 min (Figure 4c). The CPs are found on top of the NWs and are approximately 60 nm in diameter, and the NW length is nearly 2 µm. We believe that Au atoms started migrating, resulting in a smaller alloy particle compared to the 20 min growth case. Au migration should enhance NW tapering because it weakens the collection ability of the Au–In CPs according to the traditional VLS mechanism. However, our observations contradict this presumption, indicating that other processes contributing to NW growth should also be taken into account. NW morphology indicates tapering again for the sample grown for 60 min (Figure 4d). Shrinking of the Au–In CPs is evident, and the NW length increases to 3.3 µm. We also observe shorter NWs beneath these NWs, likely arising from migrated Au-catalyst-assisted growth. NWs show an increased diameter after 90 min, as shown in Figure 4e. As mentioned previously, a 2D layer forms across the substrate at a constant growth rate alongside NW growth. The 2D layer maintains a constant growth rate, while the NW axial growth rate decreases because of catalyst migration and finally stops after the CPs disappear. Furthermore, some NWs become buried under the 2D layer and cannot be distinguished in the SEM images, resulting in the shorter NWs observed at 90 min. Although 〈111〉 growth is typically dominant for Au-assisted III–V nanowires, our InSb nanowires exhibited a 〈110〉 growth axis. This arises from the specific interplay of precursor concentration, growth temperature, and Au–In–Sb droplet energetics, which stabilize {110} facets. Similar reports of 〈110〉 InSb NWs under epitaxial CVD conditions suggest that this orientation, while less common, can be reproducibly obtained under optimized growth kinetics. TEM imaging of a typical tapered NW is shown in Figure 4f–h. The NW was grown at 450 °C using a 20 mg source and a 60 min growth time, resulting in a tip diameter of 20 nm and a base diameter of 40 nm. High-resolution TEM (HRTEM) was performed to characterize the NW growth direction. A d spacing of 3.0 Å was measured corresponding to the {210} family of planes. An angular spacing of 51° was measured between the growth direction and 〈210〉, confirming a 〈110〉-type growth direction for the InSb NWs. Energy-dispersive X-ray spectroscopy (EDAX) showed that the NW body is composed of indium and antimony, while the CP at the tip of the NW also comprises Au. No Au was observed along the NW body, which might be because diffused Au served as a catalyst for nucleating smaller NWs, as shown in Figure 4d.

### 2.2. Relationship Between NW Morphology and Growth Parameters

**Figure 5:**
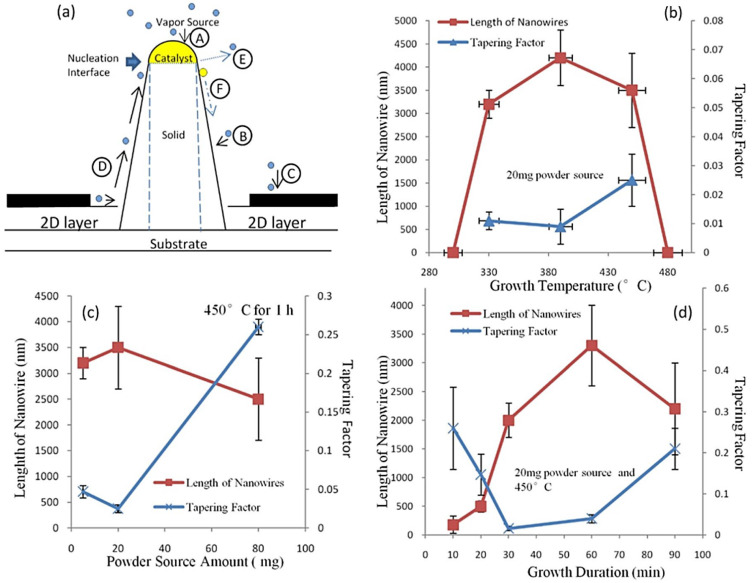
(**a**) Schematic growth model for InSb NWs: A: direct impingement of growth species from the vapor through the catalytic particle; B: direct deposition from the vapor source on the NW sidewall; C: 2D-layer deposition; D: adatom diffusion to the nucleation interface along the sidewall; E: growth-species desorption; and F: migration of catalyst (Au) atoms out of the catalytic particle. (**b**) Dependence of InSb NW length and tapering factor on growth temperature. NWs were grown with a 20 mg powder source for 60 min. (**c**) Dependence of InSb NW length and tapering factor on powder-source amount. NWs were grown at 450 °C for 60 min. (**d**) Dependence of InSb NW length and tapering factor on growth duration. NWs were grown with 20 mg of powder at 450 °C. The lines are intended as guides to the eye.

Our experiments have shown that InSb NW morphology is affected by growth temperature, the amount of powder source utilized, and growth time. Typical values of NW length and tapering factors as a function of the growth parameters are shown in Figure 5b–d, respectively. Here, the tapering factor is defined as |dbase − dtip|/L, where dbase, dtip, and L are the base diameter, tip diameter, and length of the NW, respectively. The NW morphology observed under various growth conditions can be explained in terms of the growth model illustrated in Figure 5a. In general, the model accounts for (A) direct impingement of growth species or atoms onto the catalytic particles, (B) 2D-layer deposition on the substrate, (C) diffusion of adatoms from the substrate surface toward the catalyst atoms along the NW sidewalls, (D) direct deposition on the NW sidewalls from the vapor source, (E) material desorption from the NW, and (F) migration of catalyst (Au) atoms. Influenced by the kinetics of the growth species, the NW morphology is affected by axial and radial growth modes that vary with the growth conditions.

#### 2.2.1. Growth Temperature Range and the State of Catalytic Particles (CPs)

InSb NWs are observed to grow in the temperature range of 330–450 °C. The upper growth temperature is lower than the Au–In eutectic point (454 °C) and comparable to those for InAs and InP NWs [21,68]. It has been pointed out that for Au-catalyst-assisted III–V materials growth, only group III elements dissolve in significant quantities in the seeded Au nanoparticles, forming Au–group III alloys as catalytic particles (in our case, Au–In CPs) [40,41], whereas group V elements participate in growth by diffusing through the nucleation interface. Therefore, we consider only the movement of indium and Au atoms in the discussion. We observe solid-shaped CPs at 330 °C (Figure 1b) and round, liquid CPs at 450 °C (Figure 3b). Dick et al. reported InAs NW growth by MOVPE at 420 °C, which is lower than the Au–In eutectic temperature, with solid CPs, indicating a failure of VLS growth [33]. Tchernycheva et al. suggested that Au–In CPs are liquid above 425 °C and solid below 360 °C. Although our observations are similar, we cannot simply assign a growth mechanism from the particle shape; the eutectic temperature can drop at the nanoscale, and even minor elemental contributions matter—thus, the specific ternary phase diagram should be considered. While Ghalamestani et al. [69] report that VLS growth dominates Sb-based NWs and yields uniform morphologies, our data suggest a temperature-dependent interplay. At higher temperatures, VLS dominates, yielding straight NWs; at lower temperatures, VSS contributions are likely, consistent with occasional kinking and irregularity. Thus, both mechanisms can coexist depending on the growth parameters. In general, ternary phase diagrams are complicated and there have not been enough studies on this topic so far. Furthermore, the CP phase can change with the growth temperature, gas pressure, and thermal history [70]. In situ observation and analysis of CP composition should be conducted to clarify the particle state. An in-depth study of CP phase transitions is beyond the scope of this work. Nevertheless, a clean, uniform growth direction of NWs with a solid-CP sample is shown in Figure 1b, consistent with characteristics of the VSS mechanism. Although the Au–In–Sb eutectic temperature is an important reference, prior work on InAs nanowires [24] shows that Au–In alloys can remain liquid and support VLS growth well below 480 °C. Under such conditions, reduced axial growth and enhanced radial growth are dictated by kinetic factors, including adatom diffusion and precursor incorporation rates. This suggests that in our InSb system, both thermodynamics and kinetics must be considered to explain the observed tapering and growth regime transitions. It has been reported that the VLS mechanism with liquid CPs is dominant at high growth temperatures, while the VSS mechanism with solid CPs is dominant at low growth temperatures [31]. Our observations are in agreement with this point.

#### 2.2.2. Trends of Temperature-Dependent NW Length and Tapering Factor

The typical temperature dependence of NW length and tapering factor is displayed in Figure 5b. Data were collected using a 20 mg InSb powder source and a growth time of 60 min. InSb NWs were synthesized over the temperature range of 330–450 °C. Growth at 300 °C did not produce any NWs, regardless of the amount of powder source used. Only a rough 2D layer covered the Au catalysts and microcrystals formed (Figure S2a, Supporting Information (SI)). This is because the temperature did not reach the critical threshold for NW formation. At this low temperature, the chemical potential of the source is too low to drive direct indium impingement, and the diffusion of local indium adatoms is too weak. NWs start growing at 330 °C; the NW length increases from 330 °C to 380 °C, then falls as the temperature rises to 450 °C. At 480 °C, only catalyst droplets are found after growth, with no NW growth (Figure S2b, SI). Dick et al. reported that InAs NWs cannot be grown above 480 °C, suggesting that the Au–In alloy becomes abruptly molten and no NWs can be produced above this temperature [33]. As for the tapering factor, since there are no NWs in the 300 °C and 480 °C samples, only three sets of data are presented here. The tapering factor decreases slightly from 0.011 to 0.009, then jumps to 0.025 as the temperature is raised to 450 °C. NW length initially increases with the temperature because it increases the mobility of indium adatoms on the surface. This causes them to diffuse faster to the nucleation interface, coupled with a higher rate of direct indium impingement through the CPs, and the adatom diffusion length also increases. Meanwhile, at 390 °C, the temperature is not high enough for significant Au migration; therefore, the collection ability of the CPs is not affected. All these processes enable faster axial growth than 2D-layer deposition and radial growth. In this regime, a higher temperature yields longer NWs, and the tapering factor decreases slightly with temperature.

After reaching the critical temperature, Au atoms start migrating in large quantities, which significantly weakens their collecting ability for indium atoms from the vapor. This process limits axial growth, as adatoms may diffuse to neighboring NWs at this high temperature. Because of the limited diffusion ability, these diffused adatoms reach their maximum diffusion length and nucleate on the NW sidewalls or the substrate. As a result, radial growth of NWs and formation of 2D layers are enhanced. Additionally, desorption of growth species at the NW tips is promoted as the temperature continues to increase. As a combined result of Au migration, adatom nucleation on sidewalls, and desorption, the NW length decreases with increasing temperature because axial growth is reduced. The tapering factor increases due to enhanced radial growth and 2D-layer deposition. It should be noted that at the critical temperature (approximately 390 °C in our case), although NWs exhibit a high growth rate and a low tapering factor, the curly shaped NWs (Figure 2b) are not desired. As mentioned above, complex growth kinetics at the interface cause the curly shape of NWs. We consider the critical temperature a transition temperature for NW growth because (1) Au atoms may begin migrating and the CPs are possibly in a transition state; (2) desorption from NWs may become severe from this point; and (3) supercooling of Au–In catalytic particles needs to be considered [21].

#### 2.2.3. Trends of Source Amount-Dependent NW Length and Tapering Factor

The relationship between powder-source amount and NW length/tapering factor is shown in Figure 5c. The NW length does not change significantly with the source amount. Meanwhile, the tapering factor slightly decreases from 0.047 to 0.025, then jumps to 0.26. Although direct indium impingement is limited because the CPs shrink, the NWs initially maintain a high axial growth rate owing to the considerable adatom diffusion rate.

Figure S6 plots InSb NW length versus diameter using data extracted from SEM images, where the pooled scatter reveals a modest, reproducible positive association between nanowire diameter and length (Pearson r ≈ 0.19), indicating that thicker wires are, on average, slightly longer. This trend aligns with a mechanistic picture in which diameter is largely established early (catalyst size/composition and initial alloying), while axial length reflects the time-integrated supply and interfacial kinetics (supersaturation and precursor delivery) during growth. Visual inspection suggests greater variability at larger diameters—expected as more kinetic pathways become accessible—and a clear upper-envelope trajectory, wherein the longest wires at a given diameter extend more rapidly than the median, consistent with rate-enhanced axial growth under favorable supersaturation. Occasional short/skinny points likely capture briefly interrupted growth episodes (e.g., transient catalyst depletion or local precursor dips). Because the dataset intentionally spans multiple conditions (temperature, source mass, duration), a modest global r is natural; stratifying by process window (e.g., temperature or flux) typically strengthens the observed diameter–length coupling. Collectively, these statistics highlight practical levers for uniformity: tighter seed-size distributions and stabilized droplet chemistry to narrow diameter spread, paired with stable precursor delivery and thermal control to homogenize the axial extension.

It has been mentioned that the VS mechanism can be responsible for NW growth. Considering that indium itself is a good catalyst for materials growth [71,72], it is possible that InSb NWs can be nucleated without Au assistance. Its high mobility may also enable NW radial growth by direct deposition on the grown NW sidewalls. Figure 2c and Figure 3c serve as evidence at this point, showing small, tangled NWs around the NW sidewalls and a zigzag NW morphology. Meanwhile, the 2D layer has a higher growth rate under high-PVC conditions, which causes parts of the NWs to lie beneath the layer. Therefore, we believe that under high-PVC conditions, InSb NWs have a higher propensity for radial growth, while axial growth is mainly affected by temperature. We observed similar trends in NW length and tapering factor using other source amounts (5 mg and 80 mg) and temperatures (330 °C and 390 °C) (Figure S3a–d, SI).

#### 2.2.4. Trends of Growth Duration-Dependent NW Length and Tapering Factor

The dependence of NW length and tapering factor on growth duration is shown in Figure 5d. Initially, because of the NW incubation time [67], 2D-layer deposition and adatom-diffusion-assisted growth dominate. As a result, a short NW length and a highly tapered morphology are observed. The approximately 50 nm catalytic particles also indicate that indium has not reached supersaturation, which is responsible for axial NW growth. As the growth duration increases, fully mixed Au–In CPs form. Axial growth is driven by direct indium impingement and adatom diffusion, while radial growth is driven by direct deposition on the NW sidewalls. Under the interplay of these kinetic processes, NWs exhibit a uniform diameter morphology after 30 min. Meanwhile, Au migration leads to a continuous loss of catalyst and gradually slows axial growth. It has been demonstrated that diffusing adatoms contribute to radial growth once they reach a critical diffusion length. The combined effect yields decreased axial growth and increased radial growth; therefore, NWs begin tapering again. Following this trend, Au is eventually consumed, and NWs stop growing axially. Radial growth then dominates due to adatom diffusion and direct deposition on the sidewalls. Unlike the abrupt morphology changes reported for InAs NWs with unchanged catalyst size, our samples show gradually tapered NWs, confirming that Au migration should be considered during the growth process. Overall, NW morphology is found to depend on growth temperature, source amount, and growth duration through varying axial and radial growth, governed by the interplay of multiple growth mechanisms. These trends are in good agreement with other theoretical and experimental reports [15,21,42,44,45,57].

### 2.3. Optimized Growth Conditions for InSb NWs

**Figure 6:**
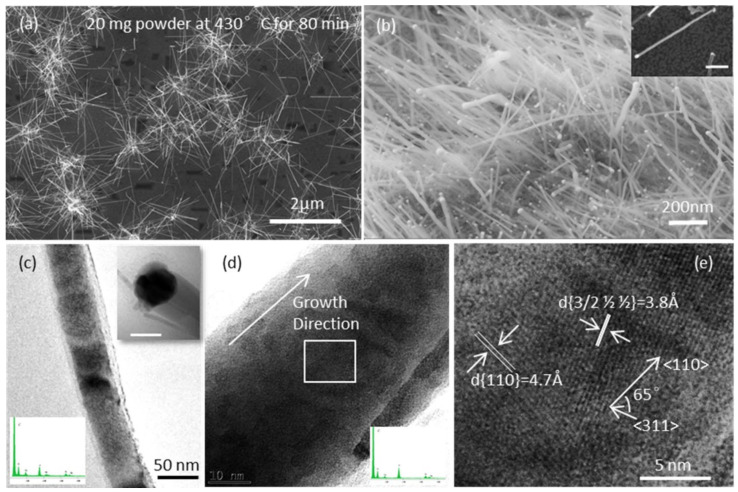
(**a**,**b**) SEM images of InSb NWs synthesized at 430 °C on an InSb (100) substrate using a 20 mg powder source for 80 min. The scale bar for the inset SEM in (**b**) is 500 nm. (**c**–**e**) TEM images of a typical NW from the as-grown sample. (**c**) TEM image of a typical tapered NW. The inset at the top right is a higher-magnification TEM of the catalytic particle; the scale bar is 50 nm. The inset at the bottom left shows the EDAX measurement of the catalytic particle region (see Figure S5 for a high resolution detailed EDAX spectrum). (**d**) HRTEM of the NW indicating the growth direction; an EDAX measurement is inset at the lower left. (**e**) A higher-magnification section of the HRTEM image in (**d**), marked with a rectangular frame. The white lines indicate measured d spacings of 3.8 Å and 4.7 Å, corresponding to the {311} and {110} planes, respectively. An angle of 65° between the growth direction and 〈311〉 confirms a 〈110〉 growth direction of the NW.

Figure 6a,b show typical top-view and corresponding tilted-view (45°) SEM images of InSb NWs with a uniform diameter along the NW body. These NWs were grown at 430 °C for 80 min with 60 nm Au catalysts using a 20 mg powder source. The NW diameters are in the range of 20–100 nm, and the length is approximately 3 µm. TEM images in Figure 6c–e reveal the detailed morphology and crystal structure of InSb NWs. Figure 6c shows that an InSb NW has a nearly uniform 50 nm diameter. A CP tip is clearly seen, as shown in the inset. HRTEM results (Figure 6c,d) show that the growth direction of the NW is along 〈110〉. The amorphous thin film around the NW is possibly due to post-growth processing. A d spacing of 4.7 Å corresponding to the {110} planes and a d spacing of 3.8 Å indicating the {311} planes are characterized in Figure 6e. A 65° angle between these two planes is observed, which is one of the interplanar angles between {311} and {110}. This confirms the 〈110〉 growth direction of InSb NWs. EDAX spectra taken from the NW body reveal no Au along the body, only at the tips. Figure S7 shows an XRD spectrum for InSb grown on an InAs substrate, consistent with 〈110〉-oriented InSb nanowires. In the zinc blende structure of InSb, the diffraction vector for the (220) family is parallel to 〈110〉; the disproportionately strong InSb (220) intensity relative to powder ratios—together with reduced (111)/(311)—indicates a 〈110〉 fiber texture, i.e., the nanowires are preferentially aligned along the 〈110〉 axis. This matches our TEM imaging, which also points to 〈110〉 axial growth.

### 2.4. Electronic Transport in Single InSb Nanowires

**Figure 7:**
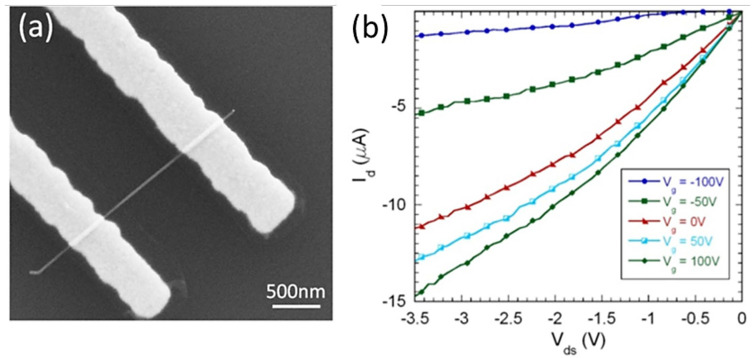
(**a**) SEM image of single 50nm InSb NW-FET. (**b**) FET source–drain current.

Electrical characterization of InSb NWs was performed using back-gated FET (field-effect transistor) devices (Figure 7a) fabricated using an isolated NW from a sample in Figure 6. The average source–drain separation was L ≈ 1 µm, and the channel diameter was ~50 nm. Room temperature I–V measurements (Agilent 4155C, Hachioji, Japan) show linear I_D_–V_DS_ over the measured bias range, indicating ohmic metal/NW contacts without thermal annealing. We attribute this to favorable Ni/InSb interfacial chemistry and small Ni grain size, which together suppress Schottky barriers at the contact. The transfer characteristic (Figure 7b) exhibits n-type conduction—consistent with slight non-stoichiometry/donor-like native defects in InSb and with prior reports on unintentionally doped III–V NWs—where ID increases monotonically with V_G_ at fixed V_DS_. From the low-bias slope of I_D_–V_DS_, we extract an average two-terminal resistance of ~250 kΩ for ~50 nm wires, and the devices sustain high current densities commensurate with the intrinsically high electron mobility of InSb. For a quantitative estimate, we outline the standard mobility extraction used for back-gated NWs. Approximating the gate capacitance by a cylinder-over-plane model, the per-length capacitance is given by [73,74],

$$C^{\prime }=\frac{2\pi \;\varepsilon_{0}\;\varepsilon_{ox}}{\mathrm{arccosh}\frac{t_{ox}+r}{r}}$$

For our geometry (Si/SiO_2_ back gate, tox ≈ 300 nm, *r* ≈ 25 nm), this gives C∼6.6 × 10^−11^ F m^−1^, so *Cg* = *C*’L∼6.6 × 10^−17^ F for L ≈ 1 μm. The field-effect mobility follows from the transconductance expression [75] in the linear regime:

$$\mu_{FE}\;=\;\frac{L}{C_{g}\;V_{DS}}\;g_{m}$$

From the I_d_–V_ds_ family, the transconductance was estimated by finite difference at |*V_DS_*| ≈ 0.5 V using ΔV_G_ = 100 V around V_G_ = 0, giving *g_m_* ≈ 25 ± 5 nS (range 20–32 nS). The back-gate capacitance was obtained from the cylinder-over-plane model, yielding Cg ≈ 66.6 aF for *L* = 1.0 µm). Using the linear-regime relation μ_FE_ = L/(C_g_V_DS_)·gm, we obtain an upper-bound field-effect mobility *μ_FE_* ≈ 7.50 × 10^6^ cm^2^/V·s. (Note that, because this estimate uses a weak back gate and a g_m_ read from an output family at finite |V_DS_|, it should be regarded as an upper bound; accurate mobility computations will require a low-bias transfer curve, refined gate capacitance evaluation, and contact de-embedding). (Details of the calculation procedures are provided in the Supplementary Information File).

Overall, these results show that as-grown InSb nanowires integrate readily into FET architectures with straightforward Ni metallization, and that contact resistance is not the primary limitation under our bias conditions—pointing instead to electrostatics (300 nm SiO_2_) and channel scattering as the dominant levers for further improvement. In future device iterations, we anticipate (i) high-k top- or wrap-gates (e.g., ALD HfO_2_/Al_2_O_3_) [76,77] to boost gate coupling and the on/off ratio, (ii) four-terminal structures to de-embed contact resistance, and (iii) gentle surface passivation prior to metallization to stabilize the native oxide and minimize interface states. These adjustments are expected to translate the favorable contact behavior observed here into higher transconductance and mobility, while preserving the simple, scalable process flow demonstrated.

## 3. Suggestions for Future Work

Future progress in controlling InSb nanowire growth will require closer integration of computation and data-driven approaches. While ground-state DFT (Density Functional Theory) has been widely applied, a major challenge is extending to dynamic, time-dependent simulations that capture evolving Au–In–Sb droplet compositions, nucleation events, and liquid–solid interfacial energetics [78,79]. Such modeling is essential because the earliest stages of nucleation dictate crystal phase, defect density, and ultimately growth morphology.

Emerging TDDFT (Time-Dependent DFT) approaches, coupled with neural network propagators, are beginning to make it feasible to study these transient processes at scale [80,81]. In parallel, AI/ML frameworks can accelerate optimization of nanowire growth by learning from structured datasets of temperature, precursor flux, and catalyst size [82]. Machine learning can uncover nonlinear correlations that govern nucleation stability and tapering, thereby guiding process windows that yield consistent and reliable morphologies with repeatable physical and electronic properties across different laboratories and manufacturing facilities.

For scalable, uniform manufacturing, these insights should be embedded into process-control strategies: (a) Incorporating real-time diagnostics (in situ TEM, RHEED, or mass spectrometry) to provide continuous feedback. (b) Using ML models trained on historical and live data streams to dynamically adjust precursor supply, substrate temperature, and carrier gas ratios. (c) Implementing closed-loop growth platforms where AI optimization ensures reproducibility across multiple reactors and wafer-scale substrates. Overall, by combining atomistic modeling (DFT/TDDFT for nucleation and interfacial energetics) with data-driven ML control frameworks, a path toward predictive design, reproducible growth, and eventual scale-up of nanowire manufacturing becomes tangible.

## 4. Summary and Conclusions

To summarize, we have systematically studied the growth of Au-assisted InSb NWs by CVD. The dependence of NW morphology (NW length and tapering factor) on a variety of growth parameters, including growth temperature, InSb powder source amount, and growth duration, has been discussed. An optimized growth condition for long and straight InSb NWs was finally achieved. It is observed that growth temperature highly influences the atoms’ direct impingement, adatoms diffusion ability, and desorption rate, which govern the NW axial growth. Meanwhile, radial growth is significantly affected by the source amount due to the vapor source’s direct deposition on the NW sidewalls and the growth substrates. Adatoms also participate in radial growth after they reach the farthest diffusion length. By analyzing the change in InSb NWs morphology with the growth duration, it shows that the catalyst Au migration should be considered when discussing the growth mechanism. We believe that the InSb NWs growth is subject to the interactions of several growth mechanisms under different conditions. Some growth mechanisms may govern the process under certain conditions. For example, the VS mechanism probably dominates the growth when the source is exceeded. VSS or VS mechanisms may be dominant at low temperatures, while VLS is dominant at high temperatures. Our results provide evidence elucidating the nature and useful information regarding the effect of growth parameters on the growth of InSb NWs. This study can be applied to gain a better understanding of the III-V NW growth mechanism and further control the growth of III-V NWs.

In conclusion, indium antimonide (InSb) nanowires are highly valued for their exceptional electrical properties, including an extremely high electron mobility, strong spin–orbit coupling, and a narrow band gap [83,84]. These characteristics make them a promising platform for future technologies, particularly in areas where conventional semiconductors like silicon fall short. In the near term, InSb nanowires could be used in high-speed, low-power transistors and sensors. Looking ahead, the integration of atomistic modeling (DFT/TDDFT) with AI/ML-driven optimization holds the potential to not only unravel the fundamental nucleation mechanisms in III–V nanowires but also to enable predictive, reproducible control over morphology. Such advances could accelerate the scalable manufacturing of uniform InSb nanowires, positioning them as key building blocks for next-generation quantum devices, high-speed electronics, and integrated photonic systems.

## 5. Experimental Section

### 5.1. InSb Nanowire Growth (CVD, Two-Tube Configuration)

*Substrates and pre-clean:* Single-side-polished InSb (100) substrates (5 mm × 5 mm) were used. Native oxide was removed by wet etch in HCl:H_2_O = 1:10 (freshly prepared), followed by sequential acetone and isopropanol (IPA) rinses. Substrates were dried under filtered Ar and loaded promptly to minimize re-oxidation.

*Au colloid deposition:* Commercial 60 nm Au colloids (Ted Pella, Inc., Redding, CA, USA) were diluted in IPA to 10^4^–10^8^ mL^−1^ particles to minimize particle–particle interactions. Dilute suspensions were dispensed and spin-coated to form a sparse, quasi-Poisson distribution of seeds; films were gently air-dried before furnace loading.

*Furnace and double-tube configuration:* Growths were performed in a horizontal quartz tube furnace using a small inner quartz tube (Ø ≈ 1 cm) inserted concentrically into the main tube to locally increase precursor partial pressure near the substrates (Figure S1, SI) [19,51,55]. InSb source powder (Alfa Aesar, 99.99%) and seeded substrates were placed at controlled separations within the inner tube to sample distinct temperature zones (source upstream, substrates downstream). Ultra-high-purity Ar/H_2_ was metered with calibrated mass-flow controllers (MFCs).

*Anneal and carrier gas:* After an Ar purge, substrates were annealed at 380 °C for 10 min under Ar + H_2_ (1:1) to remove residual solvent, stabilize Au colloids, and promote Au–In wetting during subsequent growth.

*Growth parameters:* Unless noted, total carrier flow was 75 sccm with Ar:H_2_ = 1:1 (near-atmospheric pressure). The furnace center was ramped to 570 °C at 30 °C min^−1^; the substrate-zone temperature (reported herein) covered 300–480 °C as measured by a probe positioned adjacent to the coupons. InSb source mass was varied (5, 20, 80 mg) to tune precursor vapor concentration. For each temperature–source pair, growth durations of 10, 20, 30, 60, and 90 min were explored. Each run ended with a rapid cool-down to room temperature under carrier flow before removing the inner tube. The 330–450 °C window reproducibly yielded nanowires with temperature- and precursor-dependent morphology (length/tapering), whereas 300 °C produced 2D films/microcrystals and ≥ 480 °C yielded catalyst droplets without axial growth.

*Practical notes:* Prior to the growth series, MFC setpoints were verified, and the substrate temperature reading was cross-checked against a second thermocouple placed at the coupon location. Quartz boats (Min Sheng Technology Co., Taoyuan, Taiwan) and inner tubes were pre-baked to reduce residual moisture/oxygen.

### 5.2. Post-Growth Handling

As-grown substrates were stored in dry containers. For dispersion, chips were briefly sonicated in IPA (1–2 min) to release nanowires; drops of the suspension were cast on analysis substrates: Si/SiO_2_ (300 nm) for SEM/FET and lacey carbon Cu grids for TEM. Drying proceeded at ambient conditions. For device fabrication, chips were handled with non-metallic tweezers and stored in desiccators to limit adventitious carbon/oxide growth.

### 5.3. Morphology and Structural Characterization

*SEM Imaging:* Plan-view and tilted SEM imaging used a LEO 1550 field-emission SEM (Cambridge, UK). Tip and base diameters (d_tip_, d_base_) and lengths (L) were extracted from high-magnification micrographs; the tapering factor was computed as (d_base_ − d_tip_/L). Image analysis was performed with standard measurement software to ensure consistent cursor placement and scale calibration.

*TEM/HRTEM/EDX Imaging and Spectra:* Bright-field TEM and HRTEM were performed on a Titan Themis 300 (Thermo Fisher Scientific, Waltham, MA, USA) at 300 kV equipped with EDAX (Eindhoven, Netherlands). Zone axes and lattice spacings were identified from HRTEM and FFTs/SAED. Compositional EDX confirmed In/Sb along wire bodies and Au/In in catalytic particles; within the EDX detection limit, no Au signal was detected along nanowire sidewalls under the conditions yielding straight/tapered wires. Care was taken to minimize beam-induced damage (reduced dwell, intermittent blanking).

### 5.4. Electrical Device Fabrication (NW-FET)

*Substrate and gate stack:* Devices were fabricated on p^+^ Si/SiO_2_ (300 nm) wafers (global back-gate configuration).

*Patterning and surface prep:* Dispersed nanowires were located by SEM/optical imaging. Electron-beam lithography defined source/drain pads with channel lengths ≈ 1 µm. Prior to metallization, samples received a brief O_2_ plasma clean to remove resist residues, followed by a 0.01% HF dip (seconds) to desorb native oxide; samples were promptly dried under N_2_ and loaded into the evaporator to limit re-oxidation.

*Metallization:* Contacts were Ni deposited by e-beam evaporation (typical thickness of 80–150 nm; base pressure in the 10^−6^–10^−7^ Torr range). No post-metal anneal was used.

*Electrical measurements:* I–V and transfer curves were recorded at room temperature using an Agilent 4155C (Agilent Technologies, Santa Clara, CA, USA). Devices with ~50 nm diameter nanowires exhibited linear I_D–V_DS and n-type transfer characteristics; a representative average device resistance was ~250 kΩ.

## Data Availability

The data presented in this study are available on request from the corresponding author.

## Supplementary Materials

The following supporting information can be downloaded at: https://www.mdpi.com/article/10.3390/nano15181436/s1.
