# Supplementary material for: Epitaxial Growth Control of Crystalline Morphology and Electronic Transport in InSb Nanowires: Competition Between Axial and Radial Growth Modes
Source: Nanomaterials (Basel). 2025 Sep 18;15(18):1436. doi: 10.3390/nano15181436 (PMC12472931; doi:10.3390/nano15181436)
Supplement: Supplementary file 1 [file nanomaterials-15-01436-s001.zip › nanomaterials-3848332-supplementary.pdf]

Supporting Information

Epitaxial Growth Control of Crystalline Morphology and Electronic Transport in InSb Nanowires: Competition Between Axial and Radial Growth Modes

Jiebin Zhong[1], Jian Lin[2], Miroslav Penchev[3], Mihrimah Ozkan[4]*, and Cengiz S. Ozkan[5]*

[1]Aixtron Corporation, 1700 Wyatt Drive, Santa Clara CA, 95054
[2]Department of Mechanical and Aerospace Engineering, University of Missouri, Columbia, MO 65211
[3]Center for Environmental Research and Technology, University of California, Riverside, 92521
[4]Electrical and Computer Engineering Department, University of California, Riverside, 92521
[5]Mechanical Engineering Department, University of California, Riverside, 92521

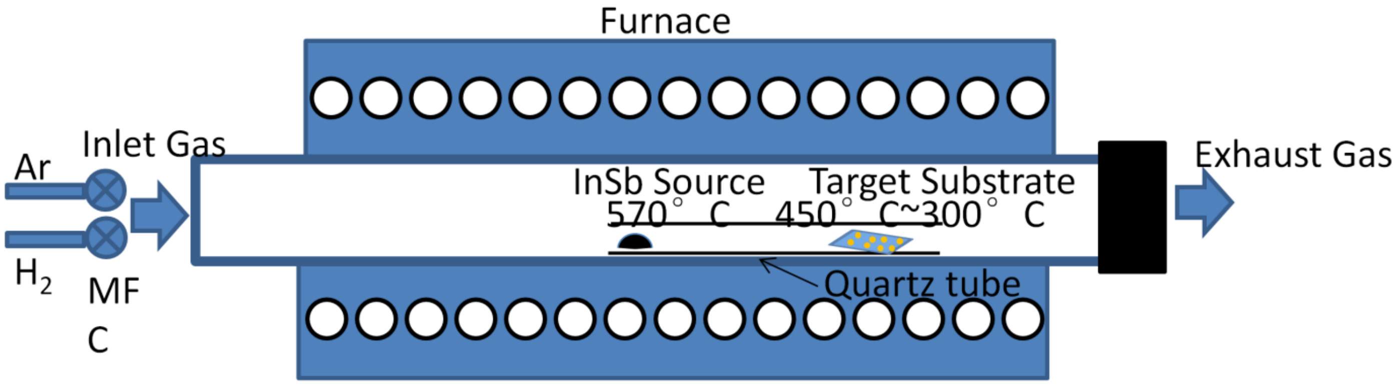

Figure S1. Schematic illustration of the experimental setup for InSb NWs synthesis by chemical vapor deposition.

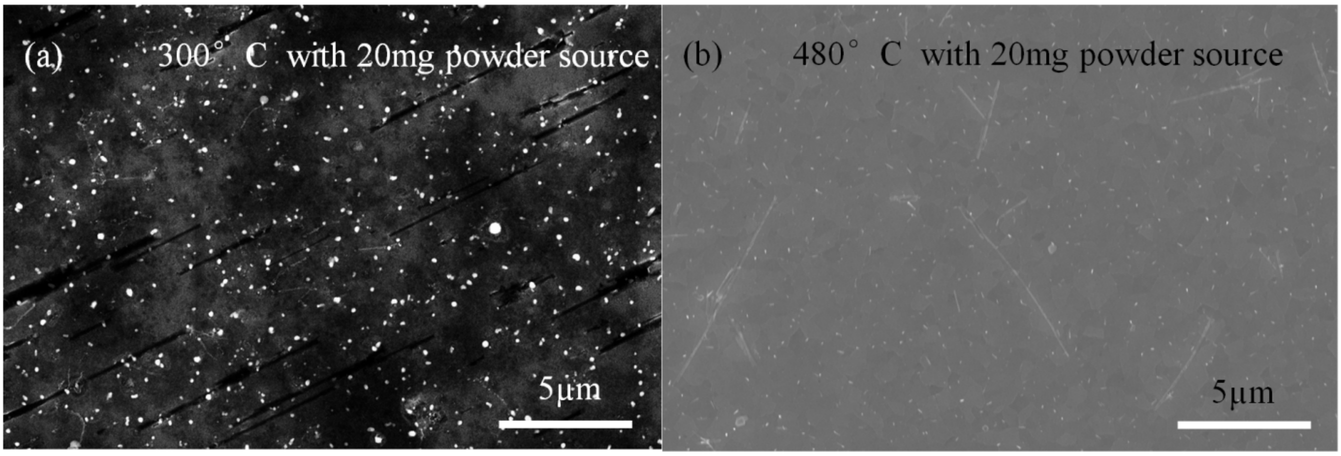

Figure S2. SEM images of InSb NWs grown at (a) 300C and (b) 480C with 20mg source.

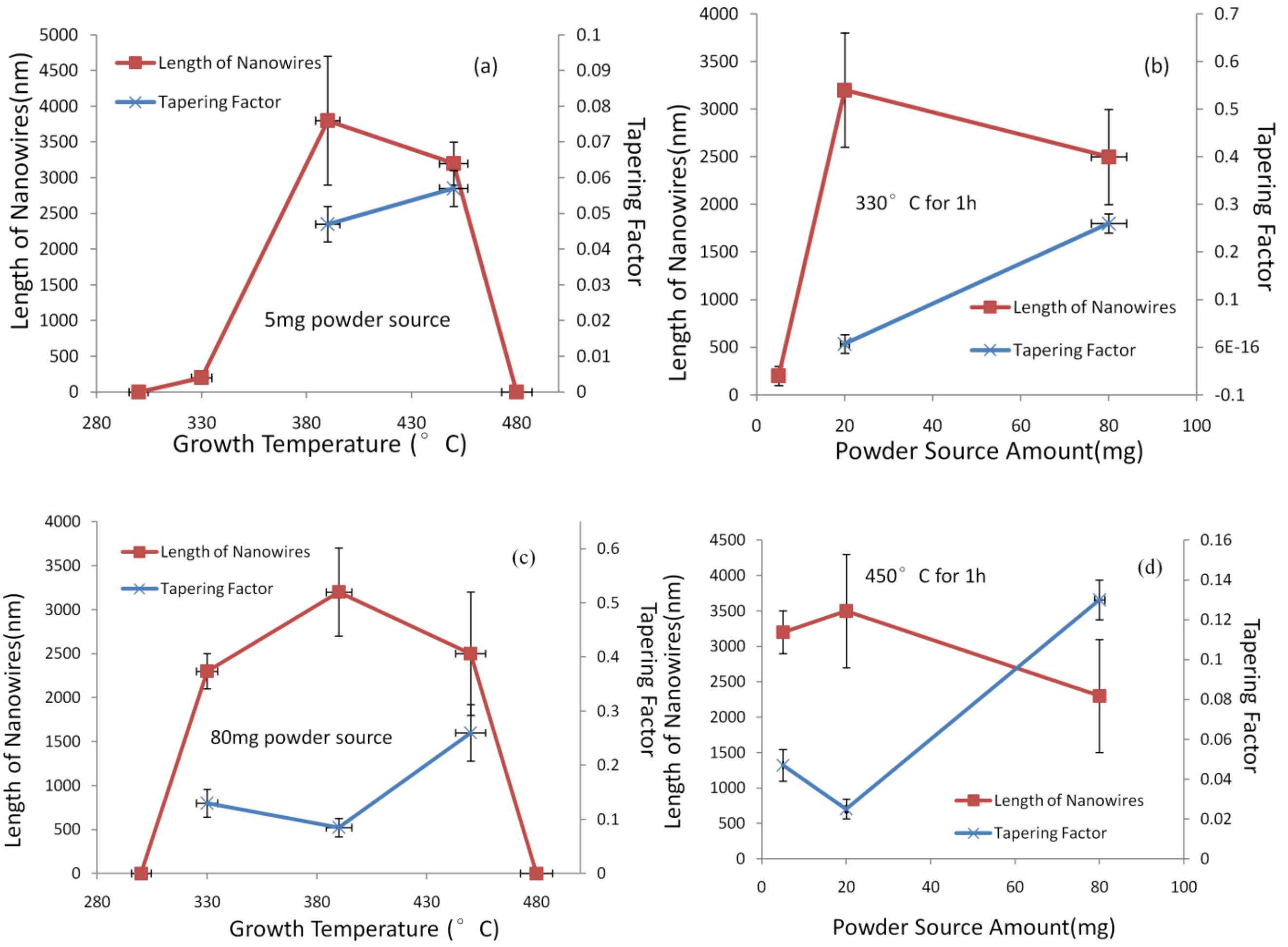

Figure S3. Dependence of InSb NWs length and the tapering factor on the temperature with (a) 5mg and (c) 80mg powder source. Dependence of InSb NWs length and the tapering factor on the source amount at (b) 330C and (d) 450C. The lines are intended as visual guides.

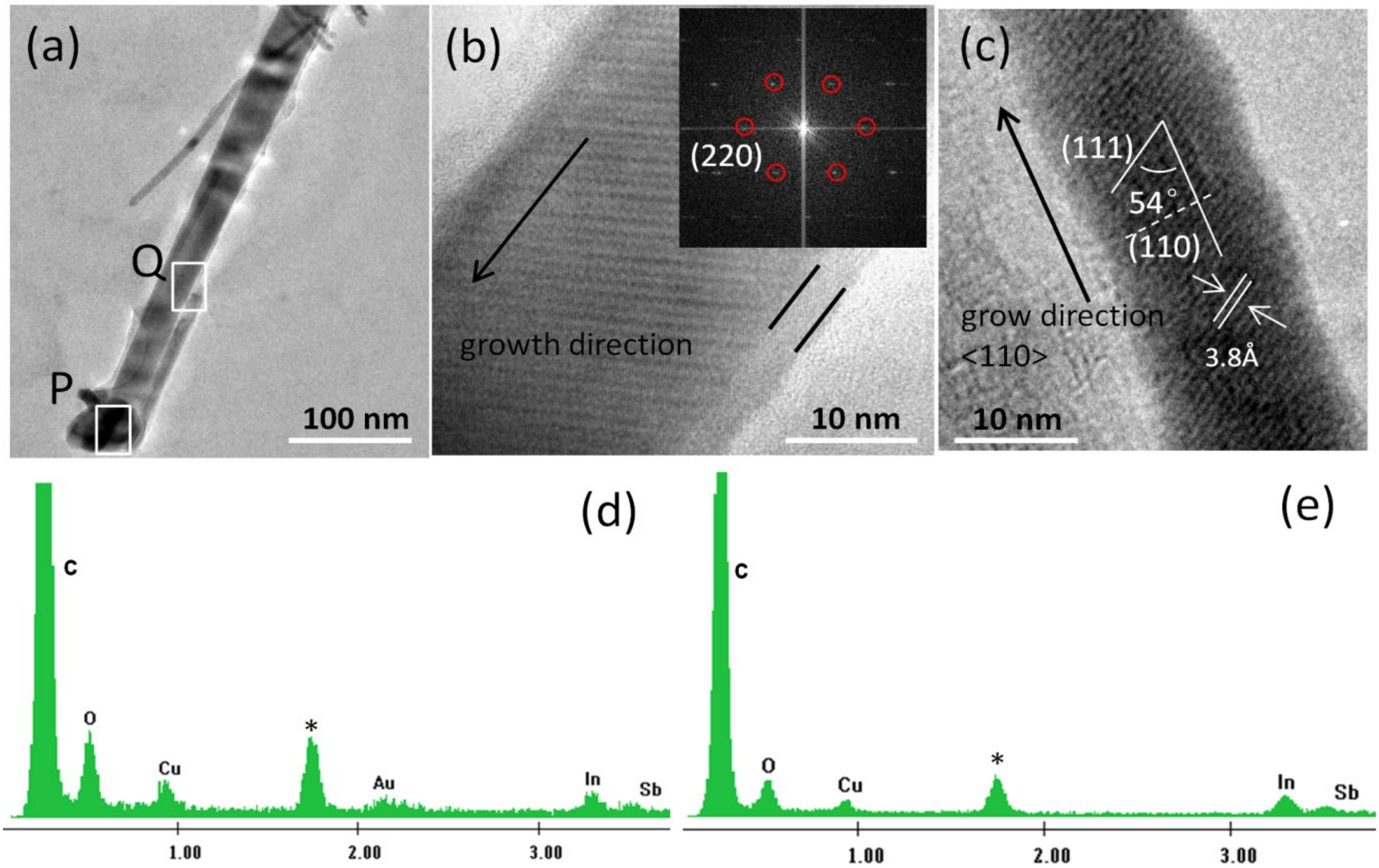

Figure S4. (a) Typical TEM and (b) HRTEM images of 30 nm InSb NWs. The inset in (b) is Fast Fourier Transform (FFT) of the TEM image showing the (220) planes of InSb zincblende structure. The oxidation layer was shown. (c) HRTEM of a 10 nm NW. The while lines show the measured d-spacing of 3.8Å, indicating {111} planes. An angle of ~36° between the growth direction and <111>, confirms the <110> growth direction of NW. (d) and (e) Respective EDAX measurements taken from the part of P and Q framed in (a), respectively. Peaks designated with '*' in the EDAX spectra are mostly likely the artifact portion during the sample preparation.

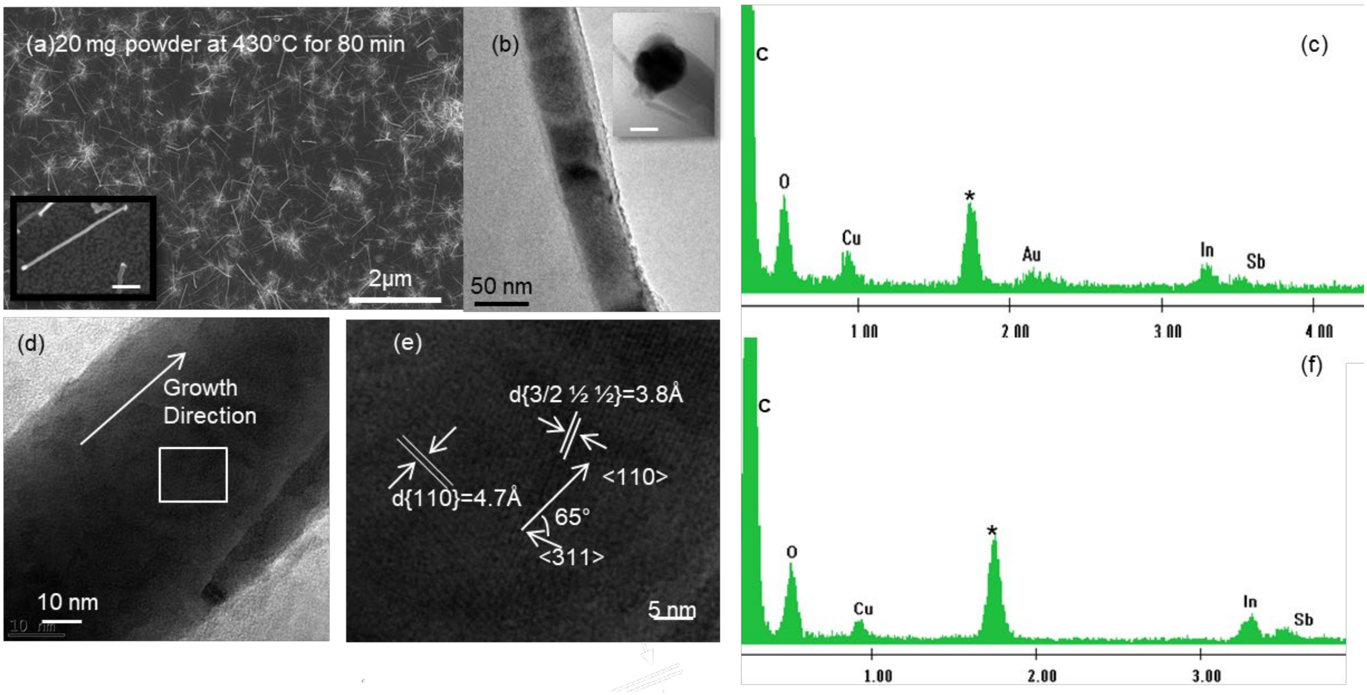

Figure S5. (a) SEM, (b) TEM, and (d), (e) HRTEM images of InSb NWs grown in optimized condition. The inset in (a) is Higher magnification SEM image of the NW with Au tip observed on top. The scale bar is 200nm. (b) shows minimally tapered NW with ~50nm in diameter. The inset in (b) is the Au tip portion the NW. The scale bar is 20nm.The corresponding EDAX of the inset is shown in (c).The white line markings in (e) show the measured d-spacing of 3.8Å and 4.7 Å, indicating {311} and {111} planes respectively. An angle of 65° between the growth direction and <311>, confirms the <110> growth direction of NW. (f) EDX measurements taken from the part framed in (d). '*' in (c) and (f)is mostly likely the artifact portion during the sample preparation.

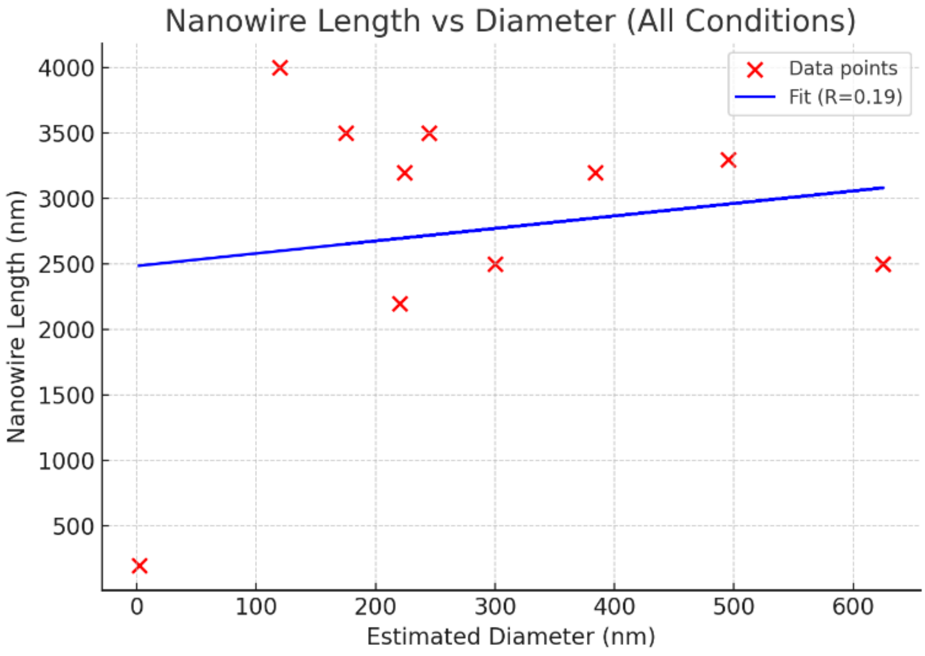

Figure S6. A plot of NW length vs diameter. Key findings from the figure include the following:

(a) The estimated correlation coefficient (R ≈ 0.19) shows a weak but positive trend: nanowires with larger diameters tend to be somewhat longer.

(b) At longer growth durations (e.g., higher powder load or longer times), the spread of both lengths and diameters increases, consistent with the reviewer's expectation for Ostwald ripening.

(c) Several points also show shorter/arrested NWs with smaller diameters, which aligns with droplet depletion by ripening.

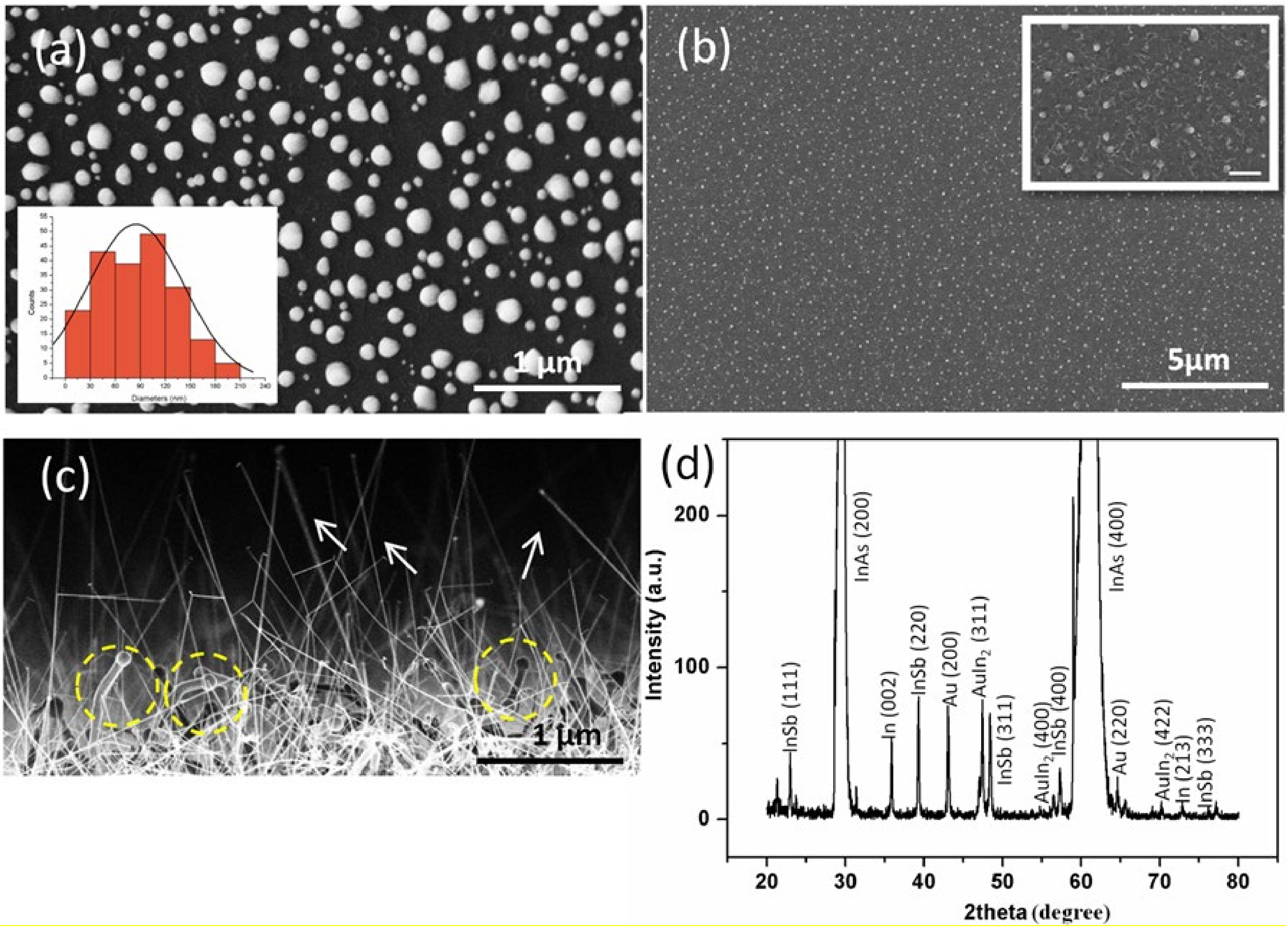

Figure S7. (a) An SEM image of Au nanoparticles dispersed on the substrate after annealing of a 50 nm thick Au thin film. The inset is the histogram of nanoparticle size distribution, ranging from 10-200nm. (b) Top view SEM image of InSb NWs grown at 430°C for 45min. The inset is a higher magnification SEM image. The scale bar is 1µm. (c) Cross-section SEM view of the InSb NWs on substrate. Larger but shorter NWs are circled and smaller but longer NWs are pointed out by white arrows. (d) An XRD spectra (Cu Kα) of InSb NWs on InAs: reflections from zinc-blende InAs (substrate) and InSb (NWs) are indexed; small 2θ separations produce close doublets (e.g., InSb (111) ≈ 22.4°, InAs (111) ≈ 23.9°). InSb peaks at (111)/(220)/(311) confirm the NW phase; intensity deviations from powder patterns reflect preferred orientation (TEM indicates ⟨110⟩ growth). No extra peaks from common oxides (In_2O_3/Sb_2O_3) are observed within the detection limit.

InSb Nanowire FET – Detailed g_m and μ_FE Calculations (with Extracted Data from the I-V plots)

A. Constants and Device Geometry

Quantity	Symbol / Value	Units
Vacuum permittivity	$\varepsilon_0 = 8.854187817\times10^{-12}$	$F\cdot m^{-1}$
Oxide relative permittivity	ε_ox = 3.9 (SiO_2)	—
Oxide thickness	t_ox = 300	nm
Nanowire radius	r = 25	nm
Channel length	L = 1.0	µm
Drain–source bias (for g_m)	|V_DS| ≈ 0.5	V

B. Gate Capacitance (Back Gate, Cylinder–Over–Plane)

$$C' = \frac{2\pi\,\varepsilon_0\,\varepsilon_{ox}}{arccosh\frac{t_{ox}+r}{r}}$$

$$C_g = C'\,L$$

Numeric evaluation: (t_ox + r)/r = 13.00, arccosh = 3.2566; C′ = 6.662e-11 $F\cdot m^{-1}$; C_g = 6.662e-17 F ≈ 66.6 aF.

C. Extracted I_D at V_DS ≈ −0.5 V (from Fig. 7b)

V_G (V)	I_D (µA)
-100	0.0
-50	-0.4
0	-2.0
50	-3.0
100	-4.0

D. Transconductance g_m via Finite Difference

Centered slope around V_G = 0 V:

$$g_m \approx \frac{ID_2(+50) - ID_1(-50)}{100\ V}$$

Numeric substitution: [I_D(+50) – I_D(−50)] = (−3.0 – (−0.4)) µA = −2.6 µA;

g_m ≈ (−2.6 µA) / 100 V = −0.026 µA/V = −26 nS (magnitude ≈ 26 nS).

One-sided local slopes around V_G = 0 V:

Lower side: [I_D (0) – I_D (−50)] / 50 V = (−2.0 – (−0.4)) µA / 50 V = −1.6 µA / 50 V = −32 nS.
Upper side: [I_D (+50) – I_D (0)] / 50 V = (−3.0 – (−2.0)) µA / 50 V = −1.0 µA / 50 V = −20 nS.
Adopted value: g_m = 25 ± 5 nS (from the 20–32 nS range).

E. Field-Effect Mobility (Linear Regime, Two-Terminal Estimate)

$$\mu_{FE} = \frac{L}{C_g\,V_{DS}}\,g_m$$

Numeric substitution: L = 1.0 µm, C_g = 6.66e-17 F, |V_{DS}| = 0.5 V, g_m = 25 nS →

μ_{FE} ≈ 7.50e+06 cm^2/V·s (range for ±5 nS: 6.00e+06 – 9.01e+06 cm^2/V·s).

Note: using an I_d–V_{ds} family at finite $|V_{DS}|$ and a weak back gate inflates μ_{FE}; so should be treated as an upper-bound. A proper mobility requires a dedicated low-bias transfer curve, refined C_g, and contact de-embedding.

F. Ohmic I_D–V_{DS} Points from Reported Two-Terminal R ≈ 250 kΩ

V_DS (V)	I_D (µA)
0.02	0.08
0.05	0.20
0.10	0.40
0.20	0.80
0.30	1.20

Implied small-signal conductance G = 1/R ≈ 4.00 µS. Assuming d ≈ 50 nm and L ≈ 1 µm, the two-terminal upper-bound resistivity ρ ≈ 0.049 Ω·cm.
